# Support interventions to promote health and wellbeing among women with health-related consequences following traumatic experiences linked to armed conflicts and forced migration: a scoping review

**DOI:** 10.1186/s13690-023-01235-8

**Published:** 2024-01-16

**Authors:** Linda Jolof, Patricia Rocca, Tommy Carlsson

**Affiliations:** 1 The Red Cross Treatment Center for Persons Affected by War and Torture, Malmö, Sweden; 2 The Department of Health Sciences, The Swedish Red Cross University, Huddinge, Sweden; 3 The Department of Women’s and Children’s Health, Uppsala University, Uppsala, Sweden

**Keywords:** Armed conflicts, Internal displacement, Refugees, Women

## Abstract

**Background:**

Women exposed to armed conflicts and forced migration face significant health-related risks and consequences. Consequently, there is a need to identify and develop effective interventions that provide tailored support for them. The aim of this scoping review was to examine research evaluating support interventions promoting the health and well-being among women with traumatic experiences linked to armed conflict and/or forced migration.

**Methods:**

A scoping review of empirical studies evaluating non-pharmacologic/non-surgical interventions promoting health and well-being among adult women with traumatic experiences linked to armed, torture, and/or forced migration, identified through systematic searches in February 2022 within five databases (AMED, CINAHL, Cochrane Library, PsycINFO, and PubMed). Methodological characteristics and results were extracted and analyzed with narrative analysis using tabulations, descriptive statistics, text-based summaries, and thematization.

**Results:**

Assessment of 16 748 records resulted in 13 included reports. The methodological approaches were quantitative (*n* = 9), qualitative (*n* = 2), and mixed methods (*n* = 2), with most reports being feasibility/pilot studies (*n* = 5) and/or randomized controlled trials (*n* = 4). The most common recruitment strategy was non-probability sampling (*n* = 8). Most interventions were conducted in North America (*n* = 4), Asia (*n* = 3) or Middle East (*n* = 3). Thirteen intra-intervention techniques and five categories of components utilized within the interventions were identified, the most common being skill building (*n* = 12). Ten developed the interventions through theoretical frameworks or manuals/therapy, while five developed interventions through public or stakeholder involvement. Eleven studies evaluated outcomes related to psychological health, disorders, or distress. A large proportion of the investigated outcomes showed post-exposure improvements and improvements when compared with controls. Qualitative findings highlighted improved mental and physical health, empowerment and stigma reduction, and enhanced knowledge.

**Conclusion:**

Few studies have developed and evaluated tailored support interventions for this population, containing a range of components and intra-intervention techniques. No clear focus was identified regarding outcome measures, and most studies used non-probability sampling. Few developed interventions through public contribution in collaboration with women. While limited studies show promising effects on women’s mental health, more empirical intervention research that closely corresponds to women’s needs are needed.

**Supplementary Information:**

The online version contains supplementary material available at 10.1186/s13690-023-01235-8.

## Introduction

A significant proportion of the global population consists of displaced women exposed to armed conflicts and forced migration, with 47 percent of the more than 82 million forcibly displaced persons being women and girls [1]. These women face several significant health-related risks and consequences, carrying a range of unique clinical profiles not necessarily represented within the corresponding male population. Women refugees report higher levels of depression, anxiety, and somatization, while being more likely to have experienced family violence and sexual abuse [2]. Research show that forced migrant women are at an increased risk of experiencing a wide range of mental health burdens [3–7], communicable and non-communicable diseases [7–10], and obstetric complications [7, 11–14]. In addition to the general dangers and challenges encountered by displaced persons regardless of their gender, women encounter significant gender-specific challenges and face a lack of services providing basic health care for women. Gender-based violence, including sexual violence, towards displaced women is a serious and prevalent issue with considerable risks and health-related consequences [15, 16]. Herein, we adhere to the definition of trauma stated by SAMHSA: “*Individual trauma from an event, series of events, or set of circumstances that is experienced by an individual as physically or emotionally harmful or life threatening and that has lasting adverse effects on the individual’s functioning and mental, physical, social, emotional, or spiritual well-being*” [17]. Within this review, we consider trauma when linked to armed conflicts, torture, and/or forced migration; meaning that the traumatic event/s could have taken place before, during, and/or after forced migration.

Many women with experience of armed conflicts and forced migration encounter unmet health needs and health-related structural inequalities, including a lack of access to health services in the host country society [9, 13, 18]. While a growing body of literature has reported health-related consequences and challenges of forced migrants in general, there is a paucity of evidence that effectively capture the specific and diverse support needs represented among specific subgroups [19]. Support interventions have the potential to strengthen women with traumatic experiences linked to armed conflicts, torture, and/or forced migration by enhancing their resilience and treat diseases or disorders. Indeed, leading organizations advocate a need to address the multidimensional health disparities and psychosocial distress observed among migrant women with traumatic experiences [20, 21]. However, a general underrepresentation of refugee women in research has been highlighted [22].

Specific knowledge about refugee women’s circumstances and lived experiences is needed, in order to successfully develop and test complex interventions tailored for subgroups of displaced persons [23]. According to a recent scoping review investigating the health of conflict-induced internally displaced women in Africa, policy interventions need to focus on developing comprehensive health intervention programs that will improve access and utilization. According to the same review, such interventions have the potential to promote knowledge, perception, and willingness among women to utilize available health services [24]. However, inconsistencies in the reporting of research testing health and psychosocial interventions hinder firm conclusions about their effectiveness and feasibility [25]. Taken together, there is a need to identify and map the breadth and characteristics of intervention research supporting these women. Herein, support interventions are defined as services aiming to promote health and wellbeing through a non-pharmacological and non-surgical method.

Society has an undeniable responsibility to ensure adequate support for women affected by armed conflicts and forced migration, a seldom-heard group in research and impacted by structural intersectional disadvantages. In their agenda for sustainable development, the United Nations highlights achieving gender equality and empowering women, while ensuring healthy lives for all and reducing inequalities within and between countries [26]. In recent years, there seems to have been an increased number of studies developing and testing support interventions for women living in settings with armed conflicts or who are forced to migrate. Thus, there is a need for efforts to map such intervention research and gain an overarching understanding concerning its breadth and scope. Previous literature reviews have highlighted the lack of research reporting on the provision of mental health and psychosocial support interventions in areas with humanitarian emergencies [27, 28]. While such interventions show promise as methods to improve functioning and post-traumatic stress, there is limited understanding of research focusing specifically on support tailored for women living under these conditions [29].

The primary aim of this scoping review was to examine research evaluating support interventions promoting the health and well-being among women with traumatic experiences linked to armed conflict and/or forced migration. A secondary aim was to map the feasibility of interventions and how interventions have been received. Specifically, the following research questions were addressed:

1. What are the methodological characteristics of the studies that have evaluated support interventions?
2. What are the components and intra-intervention techniques of the interventions, how have they been developed, and what has been reported regarding their feasibility?
3. Which health-related outcomes have been evaluated, and what effects have been reported?

## Methods

### Design

This was a scoping review of empirical studies evaluating a support intervention. This review is reported according to the PRISMA extension for scoping reviews (PRISMA-ScR) (Additional file 1) [30]. Scoping reviews are utilized to characterize and map research published within a certain topic, aiming to provide an overarching understanding of how research has been conducted in that topic [31]. Scoping reviews are appropriate when intending to systematically explore and describe breadth within a field of research. In contrast to many other systematic reviews, quality appraisals and risk of bias assessments are typically not performed in scoping reviews, based on the exploratory and mapping nature [32]. A protocol was developed a priori by the research team, which is presented in Additional file 2.

### Search methods

Pre-planned systematic searches were performed in February 2022 utilizing the five databases AMED, CINAHL, Cochrane Library, PsycINFO, and PubMed. Through joint discussions and pilot searches, final search terms were identified. Boolean operators and truncations were utilized to expand the searches. All research team members were involved in the identification of search terms, and a librarian was consulted about the search strategy before conducting the searches. In line with current recommendations for scoping reviews [32], the final search string utilized in the searches was designed in line with PCC mnemonic (population, concept, and context), including search terms related to women, forced migration, armed conflicts, torture, treatment/therapies, and intervention research (Additional file 3). Additional manual screening was performed by inspecting the reference lists in the included reports and by searching through lists of citations in the databases. Because we aimed to generate knowledge about studies published in scientific journals, no grey literature was included in this review.

### Inclusion criteria and limitations

To be included, reports needed to meet the following criteria and limitations: (1) present a quantitative, qualitative, or mixed methods scientific evaluation of an intervention in an empirical study; (2) written in English; (3) published 2012 or later; (4) investigate any health-related outcomes when exposed to an intervention following traumatic experiences of armed conflict, torture, and/or forced migration; (5) include adult women (18 years or older) with any kind of health-related consequence related to traumatic experiences linked to armed, torture, and/or forced migration; (6) evaluate any kind of non-pharmacological and non-surgical intervention aiming to promote health and well-being in the target population; and (7) be based on primary research published as an article in a scientific journal. Reports not adhering to the aforementioned criteria and limitations were excluded (Table 1). Based on the scoping nature of this review, no studies were excluded because of low methodological quality in the reporting. No filters were applied when conducting the searches.

**Table 1:** Inclusion and exclusion criteria

	**Inclusion criteria**	**Exclusion criteria**
Publication date	Publications published between 2012–2022	Publications published before 2012
Study design	Empirical experimental studies with quantiative, qualitative or mixed-methods evaluation of an intervention	Any non-intervention research including editorials, observational studies, descriptive studies, commentaries, case reports, reviews, and letters
Population	Adult (≥ 18 years of age) women with health-related consequences linked to traumatic experiences of war, torture and/or forced migration	Persons with voluntary and/or non-forced migration; persons younger than 18 years of age; other genders than women
Intervention	All support interventions provided with the specific intention to promote the health and well-being of the target population. Interventions delivered after traumatic experiences, including before, during and/or after migration, and during resettlement, in a host country	Any medical and/or surgical interventions
Outcomes	All outcomes self-reported by participants related to their health and/or well-being	Family-based outcomes, and organizational/system-level outcomes
Context	All countries worlwide	No exclusion criteria was applied for context
Language of publication	Publications written in the English language	Publications written in other languages than English

### Study selection

All hits in the databases were retrieved and uploaded in Rayyan, which was utilized to facilitate the screening procedure [33]. The first two authors performed the screening procedure independently and with blinding. Initially, all titles and abstracts were screened for inclusion and marked as included, excluded, ambiguous, or duplicate hit by both authors, respectively. Following un-blinding, ambiguous cases and conflicts in initial assessments were settled through discussions between the first two authors, and with the last author when no consensus could be reached. All remaining reports were extracted as full-text documents and read by the first two authors independently to assess final eligibility. Ambiguous cases were discussed with the last author until consensus was reached. Following full-text assessment, remaining ambiguous cases and conflicts between the first two authors were settled through discussions together with the last author.

### Data extraction and analysis

#### Data extraction

##### Methodological characteristics

Methodological details were jointly extracted by all authors utilizing a pre-designed tool, inspired by the data extraction tool presented by the JBI manual for scoping reviews [34]. The tool included details about the: (1) authors and year of publication, (2) overarching study design, (3) quantitative, qualitative, or mixed methods approach, (4) aims of the study, (5) allocation and number of study arms, (6) population under study and number of participants in the intervention group(s) and control group(s), (7) country where intervention was conducted, (8) country of origin among participants, (9) recruitment procedure, (10) mean and/or range of participant ages, (11) migration-status among participants, (12) inclusion and exclusion criteria, (14) type of intervention(s), and (15) duration of intervention(s). Any disagreements were settled through joint discussions among all authors until consensus was achieved. In line with current guidelines for scoping reviews [32], no appraisal of methodological quality and/or bias was performed.

##### Characteristics, content, and development of the interventions

The authors jointly produced narratives depicting the: (1) intra-intervention techniques (defined as the content and support mechanisms utilized within the intervention) and process of development of the intervention, and (2) any results related to the feasibility of the intervention(s), e.g., the recruitment procedure, adherence/retention/attrition, fidelity, and acceptability. Any disagreements were settled through joint discussions among all authors until consensus was achieved.

##### Health-related outcomes and post-exposure effects

The following results-related data were jointly extracted by the authors: (1) all quantitative outcomes/instruments measured and utilized for evaluation, (2) any results depicting the effects compared with control group(s) (categorized as in favor of intervention, in favor of controls, or no difference between intervention and controls), (3) any results depicting the post-exposure effects compared with pre-exposure measurements (categorized as in favor of post-exposure, in favor of pre-exposure, or no difference between pre and post exposure), and (4) a summary of the main results or conclusions. Any disagreements were settled through joint discussions among all authors until consensus was achieved.

#### Analysis

A narrative analysis was performed, inspired by the approach presented by Popay et al. [35]. Utilizing narratives and extracted information, we approached the data through tabulations and descriptive statistics. With an iterative process, maps and clusters depicting the content and effects of the utilized interventions in the reports were constructed. Qualitative results were analyzed with an inductive approach in which categories, defined as clusters of methods, intra-intervention components, and outcomes, were identified through a process of joint discussions and tabulations. Themes illustrated the manifest content in the reports, and thus, we strived for as little interpretation as possible during the thematic analysis. In line with current recommendations for scoping reviews [32], we conducted a descriptive qualitative analysis to provide a basic understanding of the circumstances and nuances reported about the interventions, including experiences among participants when participating in the study. The authors collaborated in producing narratives and the data extraction, leading to refinement until consensus was reached among all authors. Disagreements were settled through discussions.

## Findings

### Selection of sources of evidence

**Fig. 1:**
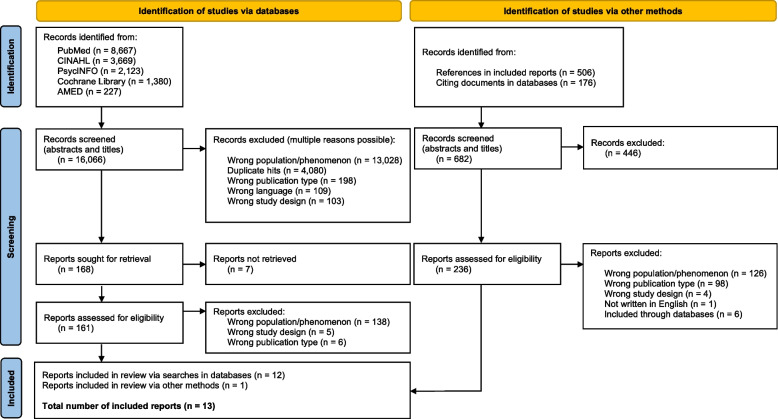
The process of searching and screening for reports

The systematic searches yielded a total of 16 066 hits, of which 15 898 were excluded based on the screening of titles and abstracts, and seven hits were inaccessible. Thus, 161 reports were more closely assessed, leading to 149 being excluded after reading full-text documents. Following the assessments, 12 reports were included through the systematic searches in databases. Additional manual searches resulted in 682 identified entries, of which 466 were excluded after screening of titles and abstracts. Following the assessment of 236 full-text documents identified through manual searches, one report was included. This resulted in 13 included reports in total (Fig. 1 [36]). The included reports are summarized in Table 2.

**Table 2:** Summary of each of the included studies (*n* = 13)

**Report, country of study (publication year)**	**Study design**	**Aim**	**Intervention, duration of intervention**	**Participants in intervention (IG) and control groups (CG), n**
Alsheikh, Jordan (2020) [37]	Quasi-experimental study	To examine the effectiveness of a group counseling program as an intervention to improve the well-being and reduce post-traumatic stress disorder among refugee women displaced due to civil wars	Group Counseling Program, 8 sessions (2 sessions per week)	IG: 20; CG: 20
Baird, et al., USA (2017) [38]	Feasibility/ pilot study	To evaluate the acceptability and feasibility of a community- based culturally tailored mental health intervention for refugee women living in a metropolitan area	Healthy Sudanese Families, 10 weekly sessions	IG: 12; CG: none
Eskici, et al., Turkey (2021) [39]	Feasibility/ pilot study and randomized controlled trial	To determine the effectiveness, feasibility, and acceptability of culturally adapted cognitive behavioral therapy for refugee women	Culturally Adapted Cognitive Behavioral Terapy, 7 weekly sessions	IG: 12; CG: 11
Hagl, et al., Bosnia (2015) [40]	Controlled trial	To compare the efficacy of dialogical exposure group treatment in the treatment of symptoms stemming from traumatic loss in post-war society	Dialogical Exposure Group using Gestalt empty-chair method, 7 weekly sessions	IG: 60; CG: 59
Hakki, Turkey (2018) [41]	Qualitative study	To explore the theatre of the oppressed as an intervention to aid refugee women in their roles and adversity-activated development after fleeing due to conflict	Theatre of the Oppressed, 5 sessions (2 sessions per week)	IG: 3; CG: none
Khan, et al., Pakistan (2019) [42]	Feasibility/pilot study	To evaluate the feasibility and acceptability of a locally adapted Group Problem Management Plus intervention for women in conflict affected settings	Group Problem Management Plus, 5 weekly sessions	IG: 59; CG: 60
Mitschke, et al., USA (2013) [43]	Quasi-experimental study	To assess the impact of a group-based financial education course on the mental health of refugee women	Financial Literacy Plus; Financial Literacy, 12 weeks (2 sessions per week)	IG: 44; CG: 21
Rahman, et al., Pakistan (2019) [44]	Randomized controlled trial study	To assess the impact of a group-based financial education course on the mental health of refugee women	Problem Management Plus, 5 weekly sessions	IG: 306; CG: 306
Robertson, et al., USA (2019) [45]	Feasibility/ pilot study	To establish the effectiveness of a group intervention in a conflict-affected setting	Health Realization, 8 weekly sessions	IG: 21; CG: 44
Shaw, et al., Malaysia (2019) [46]	Randomized controlled trial	To examine the effects of a culturally adapted Somali Health Realization intervention on coping and mental health outcomes in refugee women	Culturally Adapted Cognitive Behavioral Terapy, 8 weekly sessions	IG: 30; CG: 9
Shultz, et al., Colombia (2019) [47]	Feasibility/ pilot study	To examine the effectiveness of culturally adapted cognitive behavior group therapy among refugee women	Interpersonal counseling, unclear	IG: 59; CG: none
Tol, et al., Uganda (2020) [48]	Randomized controlled trial	To assess the effectiveness of a facilitator-guided, group-based, self-help intervention to reduce psychological distress in refugee women	Self-Help Plus, 5 sessions	IG: 331; CG: 363
Praetorius, et al., USA (2016) [49]	Qualitative study	To assess the impact of a group-based financial education course and social enterprise on the self-reported mental health of refugee women	Financial Literacy Plus; Financial Literacy, 12 weeks (2 sessions per week)	IG: 12; CG: none

### Methodological characteristics (research question 1)

Table 3 and Additional file 4 presents the methodological characteristics of the included reports, which were published between 2013 and 2022. The methodological approaches in the included studies were quantitative (*n* = 9) [37, 39, 40, 43–48], qualitative (*n* = 2) [41, 49], and mixed methods (*n* = 2) [38, 42]. Two reports evaluated the same intervention study through different methods [43, 49]. Most reports presented feasibility/pilot studies (*n* = 5) [38, 39, 42, 45, 47] and/or randomized controlled trials (*n* = 4) [39, 44, 46, 48]. Nine studies utilized one or several control groups [37, 39, 40, 42–46, 48], while four did not include any participants allocated as controls [38, 41, 47, 49]. The most common recruitment method was convenience sampling (*n* = 8) [38–40, 42, 43, 45–47], snowball sampling (*n* = 2) [45, 46], and sampling utilizing random components (*n* = 2) [44, 48]. When control/s were utilized, most studies allocated participants through cluster- or participant-based randomization (*n* = 8) [37, 39, 42–46, 48], while fewer referred to allocation based on convenience [46] or quasi-randomization [40].

**Table 3:** Methodological characteristics of the included studies (*n* = 13)

Methodological characteristics	Total reports, n [reference]
Study design	
Feasibility/pilot study	5 [38, 39, 42, 45, 47]
Randomized controlled trial	4 [39, 44, 46, 48]
Quasi-experimental trial	2 [37, 43]
Qualitative evaluation	2 [41, 49]
Controlled trial	1 [40]
Participant recruitment	
Convenience sampling	8 [38–40, 42, 43, 45–47]
Unclear recruitment strategy	3^a^ [37, 41, 49]
Snowball sampling	2 [45, 46]
Random component in the recruitment strategy	2 [44, 48]
Register-based recruitment	1 [44]
Allocation of intervention and control groups	
Cluster-randomized allocation	4 [42, 44, 45, 48]
Randomized allocation	4 [37, 39, 43, 46]
Convenience allocation	1 [46]
Non-randomized allocation depending on symptoms	1 [47]
Quasi-randomized allocation	1 [40]
Controls	
Active control group or enhanced usual care	5 [40, 42, 44, 45, 48]
Control group with treatment as usual or waitlist	5 [37, 39, 43, 45, 46]
No control group	3 [38, 41, 49]
Region where intervention was evaluated	
North America	4 [38, 43, 45, 49]
Asia	3 [42, 44, 46]
Middle East	3 [37, 39, 41]
Africa	1 [48]
Europe	1 [40]
South America	1 [47]
Region of origin among participants	
Asia	5 [42–44, 46, 49]
Africa	3 [38, 45, 48]
Middle East	3 [37, 39, 41]
Europe	1 [40]
South America	1 [47]
Migration status of participants	
Refugees	9 [37–39, 41, 43, 45, 46, 48, 49]
Internally displaced persons	2 [42, 47]
Unclear status	2 [40, 44]
Asylum seekers	1 [46]

^a^One report was a qualitative evaluation of an intervention in which participants were recruited through convenience sampling but the secondary recruitment method of study participants selected for follow-up interview is unclear

Most interventions were conducted in North America (*n* = 4) [38, 43, 45, 49], Asia (*n* = 3) [42, 44, 46] or Middle East (*n* = 3) [37, 39, 41], while few were conducted in Africa (*n* = 1) [48], Europe (*n* = 1) [40], or South America (*n* = 1) [47]. In total, the reports analyzed data based on 1 862 participants (of which *n* = 12 participated in follow-up interviews for the same intervention study). Of these, 969 participants were allocated to the intervention, while 893 were allocated as controls. When the migration status of participants was reported, nine reports focused on refugees and/or asylum seekers who originated from countries within the Middle East, Africa, or Asia [37–39, 41, 43, 45, 46, 48, 49]. Two focused on internally displaced persons in countries within Asia and South America [42, 47]. Six reports presented the range of participant ages, collectively including participants between 18–66 years of age. Nine reports presented means and medians of participant ages, ranging from 30.9 to 46 years.

### Intra-intervention techniques, treatment components, development, and feasibility of the interventions (research question 2)

#### Intra-intervention techniques and treatment components

When specified, reports described that the interventions were in part or fully led by women/facilitators/peers (*n* = 6) [39, 42, 44, 46–48], researchers (*n* = 3) [38, 41, 46], psychologists/specialists in trauma psychology (*n* = 3) [37, 40, 41], agency staff (*n* = 2) [43, 49], content experts (*n* = 1) [38], and/or students (*n* = 1) [47]. In nine reports, those leading the intervention were mentored, trained, or supervised [39, 40, 42–44, 46–49]. Three reports used translators as part of the intervention [38, 43, 49]. The reported range of the duration of the interventions ranged between 5–24 sessions, with each session having a length of 1–3 h, and the intervention being offered one or two times each week.

In total, 13 intra-intervention techniques were identified when inspecting the descriptions of the interventions as presented in the reports (Table 4): pictorial support (*n* = 4) [38, 44, 45, 48], counseling (*n* = 3) [37, 42, 47], creative arts and craft activities (*n* = 3) [41, 43, 49], behavioral activation (*n* = 2) [39, 42], cognitive restructuring (*n* = 2) [39, 46], guided imagery (*n* = 2) [38, 40], role playing (*n* = 2) [40, 45], cognitive defusion (*n* = 1) [48], culturally indicated transition rituals (*n* = 1) [39], drawing timelines (*n* = 1) [41], goal setting (*n* = 1) [44], motivational interviewing (*n* = 1) [44], and spiritual activities (*n* = 1) [38]. Five categories illustrating the utilized components within the interventions were identified: skill building (*n* = 12) [37–40, 42–49], psychoeducation (*n* = 10) [38–40, 43–49], social support (*n* = 9) [37, 40–43, 45, 47–49], discussion and practice about existential issues (*n* = 7) [37, 38, 41, 45–48], and body-mind techniques (*n* = 6) [38–40, 45, 46, 48].

**Table 4:** The components within the interventions that were evaluated within the included studies (*n* = 13)

**Components within the interventions**	**Total reports, n [ref]**
Skill building	
Total number of reports containing skill building	12 [37–40, 42–49]
Skill building in emotional regulation	8 [37, 39, 40, 44–48]
Skill building in anger management	3 [38, 39, 45]
Skill building in coping strategies [not specified further]	3 [37, 40, 44]
Skill building in parenting	3 [38, 44, 45]
Skill building in problem solving	2 [42, 44]
Skill building in psychological flexibility	2 [47, 48]
Skill building in social enterprising	2 [43, 49]
Skill building in conflict management	1 [47]
Skill building in stress management	1 [44]
Skill building in relapse prevention	1 [44]
Psychoeducation	
Total number of reports containing psychoeducation	10 [38–40, 43–49]
Psychoeducation about mental health	5 [38–40, 45, 46]
Psychoeducation about body-mind awareness/techniques	2 [39, 45]
Psychoeducation about economics/financial aspects	2 [43, 49]
Psychoeducation about emotions	2 [40, 47]
Psychoeducation in general [not specified further]	2 [44, 48]
Psychoeducation about domestic violence	1 [38]
Psychoeducation about nutrition	1 [45]
Psychoeducation about social support and resilience	1 [45]
Psychoeducation about trauma	1 [39]
Psychoeducation about treatment	1 [38]
Social support	
Total number of reports containing social support	9 [37, 40–43, 45, 47–49]
Peer support activities	5 [40–43, 49]
Sharing of personal stories	5 [37, 40, 43, 45, 49]
Sharing of feelings	3 [40, 43, 49]
Methods to mobilize/find external social support	2 [47, 48]
Discussion and practice about existential issues	
Total number of reports containing discussion and practice about existential issues	6 [37, 38, 41, 45, 47, 48]
Promotion of the ability to have compassion for self and show compassion to others	2 [45, 48]
Promotion of an understanding of hope and hopelessness, and help with finding strategies to cope with hopelessness	2 [37, 47]
Promotion of an understanding of loss and grief, and how it can trigger distress	2 [37, 47]
Promotion of understanding of role transitions, and how it can trigger distress	2 [41, 47]
Discussion of alienation and promotion of finding strategies to cope with it	1 [37]
Promotion of interaction and change within the community	1 [38]
Exercising value clarification, to promote behaviors that are in line with personal values	1 [48]
Body-mind techniques	
Total number of reports containing body-mind techniques	6 [38–40, 45, 46, 48]
Grounding and mindfulness techniques	4 [39, 45, 46, 48]
Physical exersices and relaxation	4 [38–40, 46]
Breathing exercises	3 [39, 40, 46]
Interoceptive exposure	2 [39, 45]
Strategies for improved sleep	1 [39]

#### Development

Ten reports utilized theoretical frameworks and/or a previously developed treatment manual or therapy [37, 39–42, 44–48]. Three mentioned utilizing focus groups and/or consultations with academics/researchers, health professionals, community members, and students [37–39]. Two reports, evaluating the same intervention study, described developing/refining the intervention together with agency personnel and refugees [43, 49]. One report evaluated an intervention that had been developed through pilot/feasibility studies [48].

#### Reporting of feasibility

The proportion of approached or screened participants constituting the final sample or who finalized the data collection varied within the reports. Five reported that ≥ 72% of the approached participants constituted the final sample [42, 43, 45, 46, 48], while two reported a corresponding number of ≤ 35% [39, 44]. Common reasons for non-participation or missing data included that women were unable to attend sessions [39, 44], lack of interest or time [44, 46], not permitted by family members to participate in the study [44], and migration during the study period [48].

Nine reports described relatively high, robust and/or increasing session attendance among participants [38–40, 42–46, 48]. The ways reports described attendance varied. Three reported that > 80% of the participants attended at least 3/7 [39], 3/5, or 4/5 [42, 44] sessions. One reported attrition rates of 22–24% [43] and another reported that three of five participants attended the sessions [41]. One study reported average session attendance of 68–81% [46], and another study reported that attendance in each session was ≥ 80% [48]. Qualitative findings revealed increasing attendance over time, which was considered related to women sharing positive experiences with other women in the community [38]. The same study also found that tardiness among participants was a challenge, and suggested that flexibility was necessary when conducting this research [38].

Six reports found that the interventions were acceptable and relevant, with no adverse events related to the delivered intervention [38, 39, 42, 44, 48, 49]. Identified challenges related to the feasibility and acceptability included cultural aspects [38, 49], language barriers [38, 49], difficulties answering questionnaires [38], long session duration [42], an experienced need among participants for more support than that what was offered [49], a lack of monetary incentives [42], confidentiality issues related to group-based activities [42], anxiety over product expectations [49], and differences in program content when compared with other settings [49]. Intervention fidelity was assessed in two studies, both showing satisfactory levels [44, 48]. Qualitative findings about fidelity revealed that lay helpers experienced a trust placed on them, learned to manage their own problems, and learned to adapt the necessary support skills over time [42].

### Measured health-related outcomes and post-exposure effects (research question 3)

Five categories of the investigated outcomes were identified in the reports, including psychological health, disorders, or distress (*n* = 11) [37–40, 42–48], social support or social interactions (*n* = 4) [43, 44, 46, 48], functioning and disability (*n* = 3) [42, 44, 48], psychological reactions (*n* = 2) [40, 48], and coping and development (*n* = 2) [41, 45] (Table 5). The most common outcomes within the largest category “*psychological health, disorders, or distress*” were: post-traumatic stress disorder (*n* = 9) [37, 39, 40, 42–44, 46–48], depressive disorders (*n* = 9) [38, 39, 42–48], and anxiety (*n* = 8) [38, 39, 42–47].

**Table 5:** Health-related outcomes measured in the included studies (*n* = 13)

**Health-related outcomes (Instruments)**	**Total reports, n [ref]**
Psychological health, disorders, or distress	
All reports investigating psychological health, disorders, or distress	11 [37–40, 42–48]
Post-traumatic stress disorder (PCL-C; PCL-5; PCL-6; HRQ; HTQ; IES)	9 [37, 39, 40, 42–44, 46–48]
Depressive disorder (PHQ-9; HSCL-25; HADS-D; PHQ-SADS; SCL 90-R)	9 [38, 39, 42–48]
Anxiety (HSCL-25; HADS-A; PHQ-SADS; SCL 90-R; GAD-7)	8 [38, 39, 42–47]
General mental health or psychological distress (GHQ; K6)	3 [40, 42, 48]
Individualized outcome of personal distress (PSYCHLOPS)	3 [42, 44, 48]
Psychological wellbeing (PWB; WHO-5)	2 [37, 48]
Somatization (PHQ-SADS)	1 [43]
Emotional distress (RHS-15)	1 [46]
Psychological flexibility (AAQ-II)	1 [48]
Social support or social interactions	
All reports investigating social support or social interactions	4 [43, 44, 46, 48]
Social support (MOS-SSS; MSPSS)	3 [43, 44, 46]
Positive interactions between ethnic groups (Four-point scale)	1 [48]
Functioning and disability	
Disability assessment (WHODAS)	3 [42, 44, 48]
Psychological reactions	
All reports investigating psychological reactions	2 [40, 48]
Explosive anger (Dichotomous questions)	1 [48]
Grief (GI)	1 [40]
Coping and development	
All reports investigating coping and development	2 [41, 45]
Adversity-activated development (Grid of outcomes)	1 [41]
Coping (WAYS; OMGC)	1 [45]

**Fig. 2:**
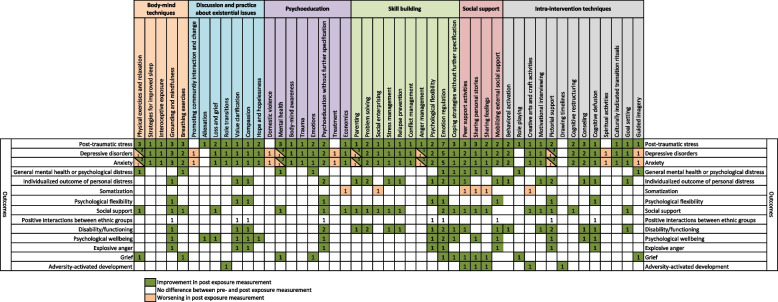
Post-exposure changes in relation to pre-exposure measurements for the investigated outcomes, presented based on the intra-intervention techniques or treatment components within the interventions (numbers in cells indicate amount of studies)

**Fig. 3:**
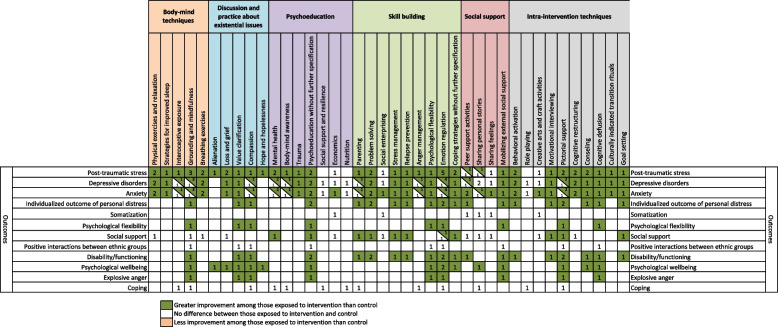
Comparisons in measurements between those exposed to intervention and controls, presented based on the intra-intervention techniques or treatment components within the interventions (numbers in cells indicate amount of studies)

Figure 2 presents the post-exposure changes in relation to pre-exposure measurements for the investigated outcomes, depending on the intra-intervention techniques or treatment components. A large proportion of the investigated outcomes showed post-exposure improvements for a range of different outcomes. Notably, post-traumatic stress uniformly showed post-exposure improvements across all identified intra-intervention techniques or treatment components. The highest numbers of studies showing improvements in investigated outcomes were found for post-traumatic stress (*n* = 7), depressive disorders (*n* = 5), and anxiety (*n* = 5), all when exposed to emotional regulation. One study showed worsened levels of depressive disorders, anxiety, and somatization, while another did not show any differences in post-exposure measurements in regard to positive interactions between ethnic groups. Figure 3 presents comparisons in measurements between those exposed to intervention and controls, depending on the intra-intervention techniques or treatment components. A large proportion of the investigated outcomes showed greater improvements among those exposed to the intervention when compared with controls. The highest numbers of studies showing greater improvements in the investigated outcomes among those exposed to the intervention were found for post-traumatic stress (*n* = 5), depressive disorders (*n* = 4), and anxiety (*n* = 4), all when exposed to emotional regulation. No study showed less improvement among those exposed to intervention when compared with controls.

The analysis of qualitative findings revealed three themes. The first theme, *improvement in mental and physical health*, illustrates the post-exposure health-related improvements. An overall improvement in mental and physical health of participants was described in three reports, including an overall sense of an improved wellbeing, happiness, hopefulness, and improved condition [38, 41, 49]. Women articulated hope after being exposed to the intervention [41, 49] and felt an enhanced ability to adjust to, or cope with, their new context [49]. One study reported that participants did not articulate similar feelings of loneliness after being exposed to the intervention [41], while another study described that the intervention involved an appreciated opportunity to keep busy [49]. In one study, participants described improvement in their headaches [49]. In two studies, participants described a general enjoyment and fulfillment related to taking part in the support intervention [38, 49].

The second theme, *empowerment and stigma reduction,* was represented in four studies which reported empowerment on an individual and community level [38, 41, 42, 49]. Participants learned new valuable skills, including cultural literacy [38, 41, 42, 49], which increased the feeling of independency, self-confidence, and self-actualization [41, 49]. Being exposed to one of the support interventions resulted in women shifting their overall perspectives and approaches to focus on positive aspects and changes [41]. Women expressed an enhanced ability to express themselves and to communicate with others, even when encountering language barriers [41, 49]. In one study, women described experiencing the environment as more humanizing after taking part in the intervention [49], while another study reported that women experienced reduced stigma and shame [38]. Participating in support interventions involved an opportunity to learn from peers [49] and made women feel more motivated to raise awareness and help others in their communities [38].

The third theme, *knowledge and information*, was represented in two studies in which women described that the support intervention resulted in enhanced knowledge, which helped them adjust to their new setting and prioritize needs over wants [38, 49]. Being exposed to the intervention resulted in better ability in recognizing symptoms of psychological distress and being more willing to seek support when needed [38].

## Discussion

### Summary of the findings

The aim of this scoping review was to examine research evaluating support interventions promoting the health and well-being among women with traumatic experiences linked to armed conflict and/or forced migration. Following systematic screenings, 13 reports were included in the review. Most studies had quantitative approaches and utilized convenience sampling. The results illustrate an overall lack of intervention research developing and testing support interventions targeted for this population. A wide range of different components and intra-intervention techniques were utilized. Although many studies evaluated outcomes related to psychological health, disorders, and distress, no clear consensus was identified regarding outcome measures. The small number of included studies suggests a lack of intervention research addressing displaced women’s support needs in general. As a scoping review, the goal was to generate an understanding of the breadth of research. Thus, the findings should not be used to inform clinical decision-making. Nevertheless, an interesting finding is that the included studies reported resounding positive post-exposure effects when compared with pre-exposure measurements and controls, highlighting a need for further systematic reviews once additional empirical studies have been conducted.

### Discussion of principal results

One research question in this scoping review was to examine the methodological characteristics of intervention research. The findings revealed a wide range of methodologies utilized within the included studies. The recruitment procedure of participants is an important aspect needing careful consideration to reach representative samples and generalizable conclusions. Sampling procedures are often complex when conducting research related to hard-to-reach or hidden populations, including migrant groups [50]. Many of the included studies utilized non-probability sampling methods, involving a risk of biased results based on over- or underrepresentation within the sample [51]. Recruitment procedures in studies investigating refugees and asylum seekers is an acknowledged challenge in need of specific efforts [52]. Often, non-probability sampling procedures are required due to practical constraints and challenges related to identifying migrant populations [50]. The utilized recruitment strategies and low sample sizes could implicate limited representativeness. On the other hand, the relatively low rejection and high retention rates suggest that the identified participants were motivated and felt a need to participate in the study. Taken together, our findings call attention to the need for identifying pragmatic and culturally sensitive approaches that can be utilized to recruit these women.

The findings highlight that research has evaluated how interventions impact women’s psychological health, disorders, or distress (e.g., post-traumatic stress and depression), experiences of social support, functioning and disability, psychological reactions (e.g., grief), as well as coping and development. Post-traumatic stress [53], depressive disorders, and anxiety [2] have been identified as major health concerns within this population, indicating that the studies addressed relevant outcomes based on observational research. On the other hand, displaced persons experience a wide range of other additional health-related consequences following traumatic events. Previous scoping reviews have also identified exposure to violence, sexual and reproductive health, other non-communicable diseases, and communicable diseases, as key health areas that needs to be addressed in future research [24, 54]. However, these challenges were not covered as outcomes in the included studies and could thus be potential valuable additional areas for intervention research to address when moving forward.

Actively engaging in public involvement and exploring prioritized research through inductive empirical research has the potential to inform researchers about relevant outcomes when conducting intervention research. Such efforts have the potential to result in tailored recruitment strategies, and further, can enhance the quality and relevancy of interventions [55]. However, the impact of collaborating with underserved populations remains unclear [56, 57] and few studies aiming to prioritize research within the refugee population have been published [58]. A small number of the included studies in our scoping review developed the interventions in collaboration with women representing the target population. This illustrates the need for research efforts that will address this gap, by utilizing and evaluating public contribution when testing interventions addressing the health and well-being among women with traumatic experiences linked to armed conflicts and forced migration. One study identified various prioritized areas not addressed in this scoping review, including how to efficiently integrate mental health support into sexual and reproductive health programs [59]. Taken together, we encourage future studies aiming to set a research priority agenda regarding support interventions for women with traumatic experiences linked to armed conflicts, torture, and forced migration.

The included studies evaluated interventions utilizing a range of different approaches, including skill building, psychoeducation, social support, discussion and practice about existential issues, and body-mind techniques. Previous reviews have made similar observations about interventions for women in conflict settings, the most commonly cited being psychosocial support and training [27]. One systematic review found a limited number of studies investigating mental health and psychosocial support interventions for populations exposed to sexual abuse and other forms of gender-based violence in the context of armed conflicts, hindering the possibility of drawing any firm conclusions about effectiveness [28]. Our results highlight the general scarcity of intervention research supporting women with traumatic experiences linked to armed conflicts and forced migration. Further, it calls attention to the wide variety of support interventions amongst the few studies included in the review. This diversity makes it difficult to draw firm conclusions about the clinical effectiveness of specific interventions. We encourage additional empirical studies followed by systematic reviews to reach conclusions that will inform clinical decision-making.

### Methodological considerations

There are methodological considerations and limitations of this scoping review. We performed systematic searches in five widely established databases and screened records through a blinded process involving two assessors. When needed, a third researcher determined the potential inclusion or exclusion of records. Additional manual screenings were conducted to identify further reports not produced through the systematic searches. While we argue that the screening procedure is robust, we nevertheless cannot disregard the potential risk that some reports could have been dismissed during the screening.

Readers should note that this scoping review aimed to provide a summary of the conducted research within this specific topic, resulting in synthesis of the breadth and characteristics of the empirical intervention research about the target population. Scoping reviews are not the most appropriate alternative to inform clinical decision-making [32, 34]. Thus, the findings should be interpreted with caution when deliberating about evidence-base of clinical praxis. In line with current recommendations for scoping reviews [32], the quantitative and qualitative analyses conducted herein were basic and descriptive. More intervention research, and subsequent systematic reviews/meta-analyses providing in-depth analyses, is necessary before it is possible to reach firm conclusions about the evidence of the effectiveness of interventions identified in this review.

We only included scientific reports written in the English language and published 2012–2022. While these criteria ensure that only recent publications were included, we cannot disregard the possibility that some relevant research was excluded. However, most records were excluded because of wrong population/phenomenon, and only a small proportion of records were excluded based on language. While five databases were utilized to search for records and references/citations were screened for inclusion, we acknowledge the risk that some research not indexed in the chosen databases was dismissed.

Women who are forced migrants with experience of traumatic events constitutes a heterogeneous population representing various backgrounds and identities. Our findings illustrate that research needs to take more consideration regarding to intersectional perspectives when conducting research providing support interventions. For example, no study included any information about women with diverse sexual orientations, gender identities, and gender expressions (e.g., lesbian, bisexual, transgender, and/or queer). Further, most participants in the included studies originated from Asia, Africa, and countries in the Middle East. These findings call attention to the need for more research including diverse samples. Readers should also note that this scoping review concerns adult women (18 years or older). We acknowledge that the definition of adulthood can vary between contexts. We encourage additional reviews addressing the health and well-being of younger women with traumatic experiences linked to armed conflicts and/or forced migration.

## Conclusion

Surprisingly few studies have developed and evaluated tailored support interventions for women with traumatic experiences linked to armed conflict, torture, and/or forced migration. Published studies have evaluated support interventions containing a wide range of different components and intra-intervention techniques, the most common being skill building and psychoeducation. In this review, no clear consensus was identified regarding outcome measures, albeit a focus on psychological health as outcome measures was observed. Participant recruitment is a challenge when conducting research addressing the health and well-being of forced migrants. Research included in this review mainly utilized non-probability sampling. It is not yet possible to draw any firm conclusions about potential clinical post-exposure effects, based on the limited studies and sample sizes as well as the lack of coherence in outcomes and intervention structure. Nevertheless, a noteworthy finding is that the limited number of included studies resoundingly reported positive post-exposure effects when compared with pre-exposure measurements and controls. The findings of this scoping review suggest that support interventions have the potential to improve the health of and knowledge among women, while empowering them and reducing stigma. We encourage researchers to continue developing and evaluating support interventions for women with health-related consequences following traumatic experiences linked to armed conflict, torture, and/or forced migration.

### Supplementary Information

**Additional file 1.** The PRISMA-ScR checklist.

**Additional file 2.** A priori protocol.

**Additional file 3.** Database searches.

**Additional file 4.** Methodological characteristics of the included reports.

## Data Availability

All data generated or analyzed during this study are included in this published article [and its supplementary information files].
