# Supplementary material for: Support interventions to promote health and wellbeing among women with health-related consequences following traumatic experiences linked to armed conflicts and forced migration: a scoping review
Source: Arch Public Health. 2024 Jan 16;82:8. doi: 10.1186/s13690-023-01235-8 (PMC10790529; doi:10.1186/s13690-023-01235-8)
Supplement: Supplementary file 2 — Additional file 2. A priori protocol. [file 13690_2023_1235_MOESM2_ESM.pdf]

PROTOCOL FOR SCOPING REVIEW

Title

Non-pharmacological and non-surgical interventions aiming to promote health and well-being among women with health-related consequences following traumatic experiences linked to war, torture, and forced migration: a scoping review

Review question

The aim of this scoping review is to provide a summary of research testing non-pharmacological and non-surgical interventions to promote the health and well-being among women with health-related consequences related to traumatic experiences linked to war, torture, and forced migration.

Specifically, the following research questions will be addressed:

1. What are the methodological characteristics of the studies evaluating the interventions?
2. What are the characteristics of the interventions that have been evaluated in research, and how have these interventions been developed?
3. What health-related outcomes have been evaluated, and what effects of these outcomes have been reported after exposure to the interventions?

Searches

Systematic searches will be performed utilizing the five databases AMED, CINAHL, Cochrane Library, PsycINFO, and PubMed. Through discussion and pilot searches, final search terms will be identified. Boolean operators and truncations will be utilized. Additional

manual screening will be performed by inspecting the reference lists in the included reports and by searching through lists of citations in the databases.

Types of studies to be included

Any empirical study published 2012 or later utilizing quantitative, qualitative, or mixed methods evaluation will be considered for inclusion. Studies need to be based on primary research and published in English as an article in a scientific journal.

Condition or domain being studied

Studies investigating any health-related outcomes when exposed to an intervention following traumatic experiences of war, torture, and forced migration will be considered for inclusion.

Participants/population

Studies recruiting adult women over the age of 17 years with any kind of health-related consequence related to traumatic experiences linked to war, torture, and forced migration will be considered for inclusion.

Intervention(s), exposure(s)

Studies testing any kind of non-pharmacological and non-surgical intervention aiming to promote health and well-being in the target population will be considered for inclusion.

Exclusion of studies

Studies not adhering to the aforementioned criteria were excluded; i.e. (1) published before 2021; (2) written in any other language than English; (3) including participants with other reasons behind their migration; (4) including persons younger than 18 years of age; (5) testing

a pharmacological or surgical intervention; (6) including participants with other genders than women; and (7) inaccessible full-text documents.

Comparator(s), control

All kinds of comparators/controls will be considered for inclusion.

Main outcome(s)

Any health-related outcome(s) will be considered for inclusion.

Additional outcome(s)

Any additional health-related outcome(s) will be considered for inclusion.

Data extraction (selection and coding)

Searches and screening of reports

1. All searches will be conducted by the last author
2. Data will be transferred to Rayyan (https://www.rayyan.ai/)
3. Any duplicates will be removed
4. The first two authors will separately screen all titles and abstracts for eligibility, blinded for each other’s assessments
5. Following un-blinding, ambiguous cases and conflicts will be settled through discussions with the last author
6. Full-text documents of the papers will be retrieved, read in full, and independently assessed for eligibility by the first two authors

7. Following full-text assessment, ambiguous cases and conflicts between the first two authors will be settled through discussions together with the last author

Data extraction

1. The following methodological characteristics will jointly be extracted by all authors: (1) authors and year of publication, (2) overarching study design, (3) quantitative, qualitative, or mixed methods approach, (4) aim of the study, (5) allocation and number of arms, (6) population under study and number of participants in the intervention group(s) and control group(s), (7) country where intervention was conducted, (8) country of origin among participants, (9) recruitment of participants, (10) mean and/or range of participant age in the sample, (11) migration-status among participants in the sample, (12) inclusion criteria, (13) exclusion criteria, (14) type of intervention(s), and (15) duration of intervention(s)
2. The following results-related characteristics will jointly be extracted by all authors: (1) all outcomes evaluated and the associated instruments utilized for measurement or evaluation, (2) any results depicting the effects compared with control group(s) for all outcomes reported (categorized as positive, negative, or no difference), (3) any results depicting the post-exposure effects compared with pre-exposure measurement or evaluation for all outcomes reported (categorized as positive, negative, or no difference), and (4) a summary of the main results or conclusions
3. The first two authors will produce narratives depicting the: (1) development of the intervention, (2) outcome-related effects, and (3) any results related to the feasibility of the intervention(s)
4. The last author will scrutinize the narratives, leading to refinement of the narratives until consensus is reached among all authors.

Risk of bias (quality) assessment

In line with the PRISMA-ScR statement and based on the aim/research questions of this review, no quality assessment will be conducted.

Strategy for data synthesis

The synthetization will be inspired by the approaches presented by Popay et al (2006). The extracted data depicting methodological characteristics will be summarized and analyzed with descriptive statistics and tabulations. Various clusterings will be performed during the analysis, including: (1) type of intervention developed and evaluated, (2) sample characteristics, (3) study design/outcomes. Qualitative results will be analyzed with inductive thematic analysis. Final summative narratives will be produced addressing each of the research questions in this review.

Contact details for further information

Tommy Carlsson

CCRN RM PHD

Associate Professor

Uppsala University and the Swedish Red Cross University

tommy.carlsson@kbh.uu.se

Organisational affiliation of the review

The Red Cross Treatment Center for persons affected by war and torture, Malmö, Sweden

The Department of Health Sciences, The Swedish Red Cross University, Huddinge, Sweden

The Department of Women’s and Children’s Health, Uppsala University, Uppsala, Sweden

Review team members and their organisational affiliations

Licensed psychologist, MSc, Linda Jolof: The Red Cross Treatment Center for persons affected by war and torture, Malmö, Sweden

Registered Physiotherapist, MSc, Patricia Rocca: The Red Cross Treatment Center for persons affected by war and torture, Malmö, Sweden

Specialist Nurse, Registered Midwife, PhD, Associate Professor Tommy Carlsson: The Department of Health Sciences, The Swedish Red Cross University, Huddinge, Sweden; The Department of Women's and Children's Health, Uppsala University, Uppsala, Sweden

Type and method of review

Narrative synthesis, Scoping review

Anticipated or actual start date

24 February 2022

Anticipated completion date

31 August 2022

Funding sources/sponsors

This review is funded by the Swedish Red Cross, Region Skåne, and Skåne Association of Local Authorities (Swedish: ”Kommunförbundet”). The funders have no role in the study design, in the collection, analysis and interpretation of data, the writing of articles, or the decision to submit for publication.

Conflicts of interest

The authors declare no conflicts of interest.

Language

English

Country

Sweden

Subject index terms

Armed Conflicts; Clinical Trial; Forced Migration; Health; Internal Displacement;

Intervention; Refugees; Scoping Review; Torture; Women

References

Popay J, Roberts H, Sowden A, Petticrew M, Arai L, Rodgers M, Britten N, Roen K, Duffy S. Guidance on the Conduct of Narrative Synthesis in Systematic Reviews: A Product from the ESRC Methods Programme. ESRC Methods Programme, 2006.
