# Supplementary material for: Support interventions to promote health and wellbeing among women with health-related consequences following traumatic experiences linked to armed conflicts and forced migration: a scoping review
Source: Arch Public Health. 2024 Jan 16;82:8. doi: 10.1186/s13690-023-01235-8 (PMC10790529; doi:10.1186/s13690-023-01235-8)
Supplement: Supplementary file 3 — Additional file 3. Database searches. [file 13690_2023_1235_MOESM3_ESM.pdf]

Database: PubMed
Search date: 2022-02-19
Complete search string: ("Psychotherapy"[MeSH Terms] OR "psychotherap*"[Title/Abstract] OR "Complementary Therapies"[MeSH Terms] OR "therap*"[Title/Abstract] OR "Internet-Based Intervention"[MeSH Terms] OR "Rehabilitation"[MeSH Terms] OR "Rehabilitation"[Title/Abstract] OR "intervention*"[Title/Abstract] OR "treatment*"[Title/Abstract] OR "therapeutic*"[Title/Abstract] OR "Clinical Trial"[Publication Type] OR "Trial"[Title/Abstract] OR "randomi*"[Title/Abstract] OR "counsel*"[Title/Abstract] OR "trauma focus*"[All Fields] OR "traumafocus*"[All Fields] OR "Mental Health Services"[MeSH Terms] OR "mental health servic*"[Title/Abstract] OR "mental health care*"[Title/Abstract]) AND ("Female"[MeSH Terms] OR "female*"[Title/Abstract] OR "Women"[MeSH Terms] OR "woman*"[Title/Abstract] OR "women*"[Title/Abstract]) AND ("Refugees"[MeSH Terms] OR "refugee*"[Title/Abstract] OR "Emigrants and Immigrants"[MeSH Terms] OR "forced migra*"[Title/Abstract] OR "immigra*"[Title/Abstract] OR "displaced person*"[Title/Abstract] OR "asylum*"[Title/Abstract] OR "Transients and Migrants"[MeSH Terms] OR "Torture"[MeSH Terms] OR "torture*"[Title/Abstract] OR "Warfare and Armed Conflicts"[MeSH Terms] OR "war"[Title/Abstract] OR "warfare"[Title/Abstract] OR "armed conflict*"[Title/Abstract] OR "gender based violence"[Title/Abstract])
Filter: published 2012-2022; English language
Hits: 8,667

Database: CINAHL
Search date: 2022-02-21
Complete search string: (MH "psychotherapy" OR ”psychotherap*” OR MH "rehabilitation, psychosocial" OR "MH internet-based intervention" OR AB “treatment*” OR TI ”treatment*” OR AB “intervention*” OR TI “intervention*” OR AB “therap*” OR TI “therap*” OR AB ”therapeutic*” OR TI ”therapeutic*” OR AB ”complmentary therap*” OR TI ”complmentary therap*” OR AB “rehabilitation” OR TI “rehabilitation” OR AB “mental health care” OR TI “mental health care” OR AB “mental health service*” OR TI “mental health service*” OR AB “clinical trial*” OR TI “clinical trial*” OR AB “trauma focus*” OR TI “trauma focus*” OR AB ”traumafocus*” OR TI ”traumafocus*” OR AB “counsel*” OR TI “counsel*” OR MH "Alternative Therapies" OR MH "clinical trials" OR AB ”trial” OR TI ”trial” OR AB ”randomi*” OR TI ”randomi*”) AND (MH "Women+" OR MH "female" OR AB “woman” OR TI “woman” OR AB "female*" OR TI “female*” OR TI "women*" OR AB "women*") AND (MH "refugees/PF" OR AB “refugee*” OR TI “refugee*” OR AB “asylum seek*” OR TI “asylum seek*” OR AB ”asylum*” OR TI ”asulym*” OR AB “immigra*” OR TI “immigra*” OR AB “displaced person*” OR TI “displaced person*” OR AB “forced migra*” OR TI “forced migra*” OR MH "residential mobility" OR MH "immigrants" OR MH "Torture" OR AB “torture*” OR TI “torture*” OR MH "Torture Survivors" OR AB “war” OR TI “war” OR AB “warfare” OR TI “warfare” OR AB "armed conflict* OR TI "armed conflict* OR AB “gender based violence” OR TI “gender based violence”)
Filter: published 2012-2022, English language
Hits: 3,669

Database: PsycINFO
Search date: 2022-02-22

Complete search string: (MAINSUBJECT.EXACT.EXPLODE("Treatment") OR (”psychotherap*”) OR (”complementary Therap*”) OR ("therapeutic*") OR ("treatment") OR ("therap*") OR ("intervention*") OR ("rehabilitation") OR MAINSUBJECT.EXACT.EXPLODE("Clinical Trials") OR ("clinical trial*") OR (”trial*”) OR ("randomi*") OR ("counsel*") OR ("trauma focus*") OR ("traumafocus*") OR ("mental health servic*") OR ("mental health care*")) AND (DE("Refugees") OR MAINSUBJECT.EXACT.EXPLODE("Asylum seeking") OR MAINSUBJECT.EXACT.EXPLODE("War") OR MAINSUBJECT.EXACT.EXPLODE("Violence") OR MAINSUBJECT.EXACT.EXPLODE("Torture") OR MAINSUBJECT.EXACT.EXPLODE("Immigration") OR ("refugee*) OR ("immigra*") OR ("forced migra*") OR ("displaced person*") OR MAINSUBJECT.EXACT.EXPLODE("Political Asylum") OR ("asylum seek*") OR (”asylum*”) OR ("torture*") OR ("war") OR ("warfare") OR (”war”) OR ("armed conflict*")) AND (MAINSUBJECT.EXACT.EXPLODE("Human Females") OR (”Female*”) OR (”Women*”) OR (”Woman*”) OR ("gender based violence"))
Filter: published 2012-2023, English language
Hits: 2,123

Database: AMED
Search date: 2022-03-15
Complete search string: ((”treatment") OR (”psychotherap*”) OR (”complementary therap*”) OR ("therap*") OR ("intervention*") OR ("rehabilitation") OR ("clinical trial*") OR (”trial*”) OR ("randomi*") OR ("counsel*") OR ("trauma focus*") OR ("traumafocus*") OR ("mental health servic*") OR ("mental health care*")) AND (("war") OR ("immigra*") OR ("refugee*) OR ("forced migra*") OR ("displaced person*") OR ("political asylum") OR ("asylum seek*”) OR (”asylum*”) OR ("torture*") OR ("warfare") OR ("armed conflict*")) AND ((”Female*”) OR (”Women*”) OR (”Woman*”) OR ("gender based violence"))
Filter: published 2012-2022
Hits: 227

Database: Cochrane Library
Search date: 2022-03-09
Complete search string: ("Psychotherapy"[MeSH] OR "psychotherap*" OR "Complementary Therapies" OR "therap*" OR "Internet-Based Intervention"[MeSH] OR "Rehabilitation"[MeSH] OR "Rehabilitation” OR "intervention*" OR "treatment*" OR "therapeutic*" OR "Clinical Trial" OR "Trial" OR "randomi*" OR "counsel*" OR "trauma focus*" OR "traumafocus*" OR "Mental Health Services"[MeSH] OR "mental health servic*" OR "mental health care*") AND ("Female"[MeSH] OR "female*” OR "Women"[MeSH] OR "woman*" OR "women*") AND ("Refugees"[MeSH] OR "refugee*" OR "Emigrants and Immigrants"[MeSH] OR "forced migra*" OR "immigra*” OR "displaced person*" OR "asylum*" OR "Transients and Migrants"[MeSH] OR "Torture"[MeSH] OR "torture*" OR "Warfare and Armed Conflicts"[MeSH] OR "war" OR "warfare" OR "armed conflict*" OR "gender based violence")
Filter: Trials; Published within 10 years
Hits: 1,380
